# External Validation of BMT-*i* Computerized Test Battery for Diagnosis of Learning Disabilities

**DOI:** 10.3389/fped.2021.733713

**Published:** 2021-10-01

**Authors:** Catherine Billard, Camille Jung, Arnold Munnich, Sahawanatou Gassama, Monique Touzin, Anne Mirassou, Thiébaut-Noël Willig

**Affiliations:** ^1^Association pour la Recherche sur les Troubles des Apprentissages, Paris, France; ^2^Clinical Research Center, Centre Hospitalier Intercommunal de Créteil, Créteil, France; ^3^Université de Paris and Imagine Institute (INSERM UMR1163), Paris, France; ^4^Centre Ressource sur les Troubles des Apprentissages, Paris Santé Réussite, Paris, France; ^5^ELSAN & EvEnTAil Assessment Center, Toulouse, France; ^6^Association Française de Pédiatrie Ambulatoire, Orléans, France

**Keywords:** BMT-*i*, test battery, screening, learning disabilities, academic skills, cognitive functions, validity, child

## Abstract

**Background:** Learning disabilities (LDs) are a major public health issue, affecting cognitive functions and academic performance for 8% of children. If LDs are not detected early and addressed through appropriate interventions, they have a heavy impact on these children in the social, educational, and professional spheres, at great cost to society. The BMT-*i* (Batterie Modulable de Tests informatisée, or “computerized Adaptable Test Battery”) enables fast, easy, reliable assessments for each cognitive domain. It has previously been validated in children ages 4–13 who had no prior complaints. The present study demonstrates the sensitivity of the BMT-*i*, relative to reference test batteries, for 191 children with cognitive difficulties.

**Materials and Methods:** These 191 subjects were included in the study by the 14 pediatricians treating them for complaints in five cognitive domains: written language [60 (cases)]; mathematical cognition (40); oral language (60); handwriting, drawing, and visuospatial construction (45); and attention and executive functioning (45). In accordance with a predefined protocol, the children were administered BMT-*i* tests first, by their pediatricians, and reference tests later, by specialists to whom the BMT-*i* test results were not disclosed. Comparison of BMT-*i* and reference test results made it possible to evaluate sensitivity and agreement between tests.

**Results:** For each of the five domains, the BMT-*i* was very sensitive (0.91–1), and normal BMT-*i* results were highly predictive of normal results for specialized reference tests [negative likelihood ratio (LR–): 0–0.16]. There was close agreement between BMT-*i* and reference tests in all domains except attention and executive functioning, for which only moderate agreement was observed.

**Conclusion:** The BMT-*i* offers rapid, reliable, simple computerized assessments whose sensitivity and agreement with reference test batteries make it a suitable first-line instrument for LD screening in children 4–13 years old.

## Introduction

The high prevalence of learning disabilities (LDs)—estimated at 8% among children ages 3–17 (1)—makes them a public health priority worldwide. LDs are neurodevelopmental disorders that impact one or more cognitive functions in affected children, who may struggle with the development of academic skills (written language and mathematical cognition), early language and fine motor skill acquisition, or maintaining attention (DSM-5) (2). Current models attempt to integrate (i) neuropsychological knowledge about learning, (ii) underlying cognitive abilities, and (iii) neurobiological aspects, including potential inheritance and environmental factors (3). Researchers are overwhelmingly in favor of early LD detection because the efficacy of rapid treatment has been demonstrated (4–8). The diversity of the domains affected, alone or in combination, requires thorough evaluation of the nature, severity, and development of deficits (2, 9, 10). The consequences of LDs on the personal, academic, and later, professional lives of children depend on how early they are treated (8). Recommendations made by the French National Authority for Health (HAS) define treatment paths for children with LDs in France according to the severity of the disorders and how quickly they progress (11). These HAS recommendations indicate the role of physicians in screening, referral to specialists, and coordination with teachers. Though countries differ in how they manage LD treatment (12), evaluation of affected cognitive domains and the progression of deficits requires carefully validated instruments in the language of the children assessed (13).

The computerized Adaptable Test Battery (BMT-*i*) is a set of tests for the first-line assessment of children's academic skills and cognitive functions, from kindergarten (age 4) to seventh grade (age 13). It permits broad exploration of written language abilities (reading fluency, reading comprehension, and spelling), mathematical cognition (numbers, arithmetic, and problem-solving), and three further cognitive domains (verbal, non-verbal, and attention and executive functioning). BMT-*i* tests are meant to be simple to administer, short (10–30 min per domain, depending on age), and easy to score, and they can be taken at school or during an appointment with a health professional. Their purpose is rapid identification of children who require specialized assessments for precise LD diagnosis (14, 15).

We recently reported the validation of the BMT-*i* for a sample of 1,074 French children with no prior complaints (15). Here we present its validation for a group of children with cognitive difficulties suggesting possible LDs. We demonstrate that the sensitivity of the BMT-*i* and its agreement with reference test batteries make it a robust tool for initial detection of LDs in children.

## Materials and Methods

### Participants

**Figure 1:**
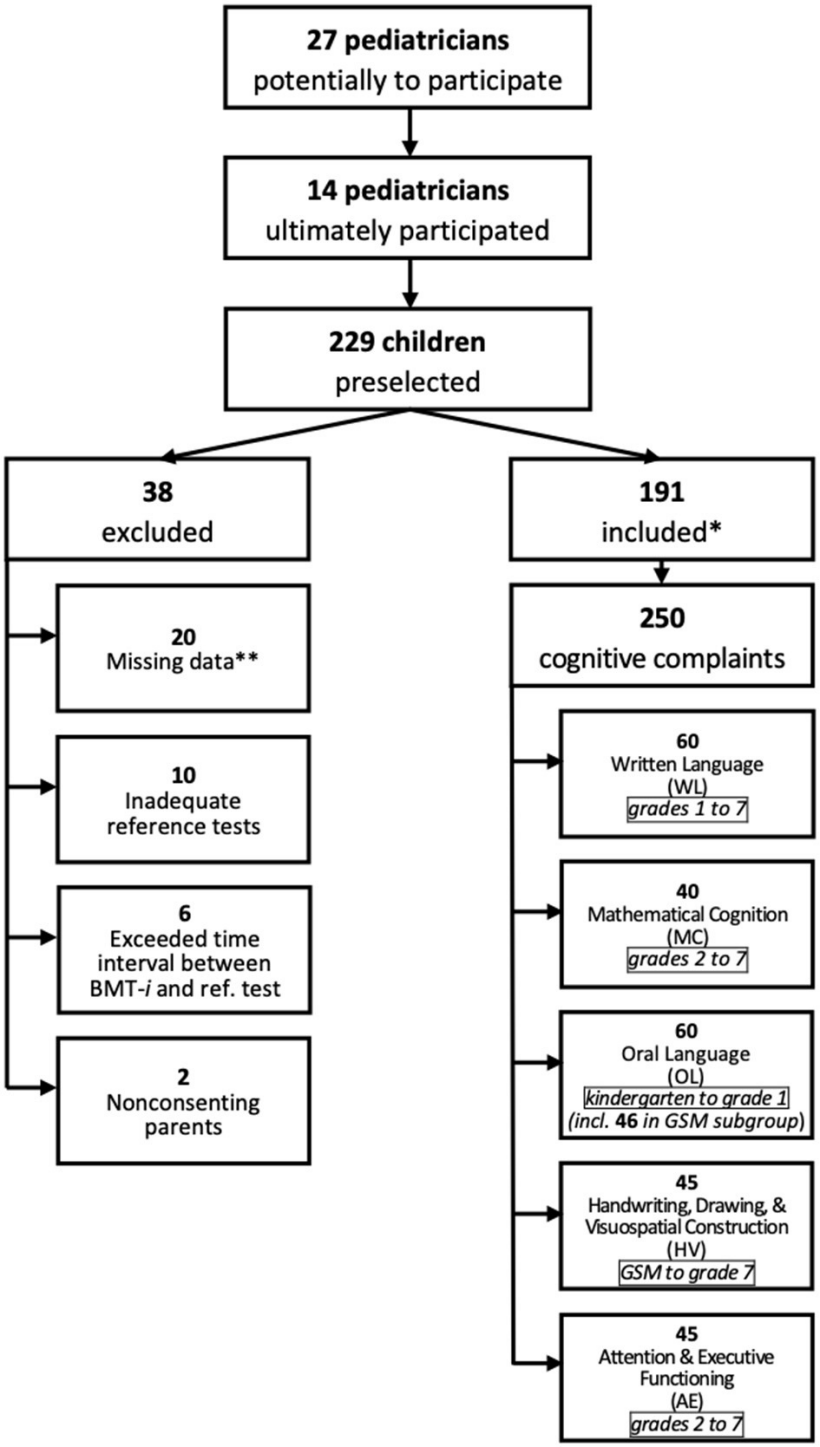
Study recruitment. *With complaints in one or more cognitive domains. **Excluded because >1 datum missing. GSM, final year of French kindergarten (*grande section de maternelle*).

The study population consisted of children suspected of having LDs due to complaints concerning one or more of the following cognitive domains: written language (WL); mathematical cognition (MC); oral language (OL); handwriting, drawing, and visuospatial construction (HV); and attention and executive functioning (AE). Child patients were recruited by 14 pediatricians at their offices or in hospitals (Figure 1). These practitioners had expertise in LDs including the use of the BMT-*i* for their professional screening practice. All pediatricians collaborating to the study received a 2-days specific training on the use of the BMT-*i* as part of the protocol. In addition, a member of the research team (who had no access to the specialized evaluations) was available to address questions.

The number of subjects included (>184 children) was calculated from the desired accuracy of 5% with a 95% confidence interval, for an expected sensitivity of 0.85 and a disease prevalence of 75% in this population with complaint. Nearly two hundred children were included, about a quarter of whom had cognitive complaints in multiple domains. Few children were lost to follow-up.

To be eligible for inclusion, children had to be (i) at least 4 years old and no more than 13 years and 11 months old, (ii) registered with the French social security system, (iii) seeing their pediatrician for a complaint, defined by symptoms their parents described, that called for a specialized evaluation within 4 months. Children known to have an intellectual disability or autism spectrum disorder, or whose both parents were not speaking French, or who had been in the French school system for <2 years, were not eligible. Pediatricians approached the legal representatives of eligible children, offering to include them in the study.

Written informed consent was first obtained from the legal representatives of children who were to be included. The study protocol was approved by an ethics committee (CPP 2018-A-O1870-55).

### Test Administration

Once consent was obtained, the children had appointments with their pediatricians, who administered the BMT-*i* tests assessing the particular cognitive domains corresponding to their complaints. Then, within 4 months, these children were reassessed by specialists uninformed of the BMT-*i* results, using reference test batteries. Study coordinators verified inclusions and protocol observance, and independently collected the BMT-*i* and reference test battery data. Table 1 presents BMT-*i* and corresponding reference battery tests, according to age and cognitive domain.

**Table 1:** Breakdown of BMT-i and reference tests used, by cognitive domain and school grade.

**Cognitive domain**	**Skills assessed**	**School grades**	**Proportion of cases**	**BMT-*i* test**	**Reference test (proportion of children tested)**
Written Language (WL)	Decoding and dictation: errors	1	5%	Decoding; dictation	
	Decoding: *time (*s*), errors* Comprehension: *accuracy* Dictation: *total errors, time (*s*)*	1–4	72%	*Couleurs magiques* (text); dictation	**EVALEO** (55%) **Exalang 5–8, 8–11, 11–15** (28%) **BELO** (17%)
				*Un beau petit vélo* (text); dictation	
		5–7	23%	*Star du Rap* (text); dictation	
Mathematical Cognition (MC)	Numerical representation: *accuracy* Arithmetic: *accuracy* Problem-solving: *accuracy*	2	25%	Numbers: reading/dictation Mental math fluency Problems	**TEDI-MATH Petits** (25%) **TEDI-MATH Grands** (50%) **Examath 8–15** (25%)
		3–7	75%		
Oral Language (OL)	Lexical production and comprehension and; syntactic production and comprehension; phonology: *accuracy*	KG	77%	Lexical production Lexical comprehension Syntactic production Syntactic comprehension Phonology	**Evalo** (77%) **Exalang 2–6, 5–8** (13%) **EVALEO** (10%)
		1	23%		
Handwriting, Drawing, and Visuospatial Construction (HV)	Copying simple figures	GSM−4	GSM−1: 11% 2–4: 68% 5–7: 21%	Simple figures: *accuracy, time (*s*)*	**VMI** (11%); **NEPSY-II** (71%): *accuracy*
	Copying complex figure	2–7		Complex figure: *accuracy, time (*s*)*	**Rey complex figure** (100%): *accuracy*
	Handwriting	2–7		Handwriting for dictated text	**BHK** (100%): *quality, time*
	Visuospatial construction	2–5		Construction: *accuracy, time (*s*)*	**NEPSY-II** (71%)—block construction **WISC-V** (93%)—block design
Attention And Executive Functions (AE)	Selective attention	2–7	2–3: 44% 4–6: 38% 6–7: 18%	Auditory attention: *accuracy for “control” task*	**NEPSY-II** (100%)—auditory attention: *accuracy*
	Sustained attention			Visual sustained attention: *% negative errors, i.e., omissions; RT, median and standard deviation (*ms*)*	**CPT 3** (5%): *% negative errors, i.e., omissions; RT, median and standard deviation (*ms*)* **KiTAP/TAP** (31%)—sustained attention: *% negative errors; RT, median and standard deviation (*ms*)* **Tea-ch** (60%)—code transmission: *accuracy*
	Inhibition/flexibility			Auditory attention: *accuracy for “conflict” task* Visual attention: *% positive errors*	**NEPSY-II** (100%)—response set **CPT 3** (5%): *% positive errors* **KiTAP** (31%): *flexibility* **Tea-ch** (64%)—*Marche-arrête, Petits hommes verts, Mondes contraires*
	Working memory	2–7		Digit span, forward and backward	**CMS**/**WISC-V**—Digit span

*GSM, final year of French kindergarten (grande section de maternelle); KG, kindergarten; RT, reaction time*.

#### Administration of BMT-*i* Tests by Pediatricians

For each participant, pediatricians recorded medical history, including perinatal data and information on prior treatment for the complaint; noted if one of the parents spoke a language other than French; and identified any financial hardship entitling the patient to free care. Parents were provided with a questionnaire they passed on to their child's teacher on which the latter rated their student's difficulties (none, moderate, or major) in each cognitive domain. During individual appointments under normal conditions and lasting 30–40 min, pediatricians administered the BMT-*i* tests corresponding to their patient's complaint and school grade (Table 1) (14, 15).

Reading (speed, accuracy, and comprehension) and spelling tests assessed WL complaints. For complaints concerning MC, number reading and dictation, mental math, and problem-solving tests were used. In the case of OL complaints, tests evaluating phonology, lexical production and comprehension, and syntactic production and comprehension were administered. For HV-related complaints, children were asked to copy simple and complex figures, where speed and quality reflected drawing abilities, and perform 15 cube construction tasks, where the same variables measured visuospatial construction skills. A handwriting score was assigned for dictations. In addition, motor skills were assessed using the European-French Developmental Coordination Disorder Questionnaire (DCDQ-FE), which detects motor skill deficits (16). When children's complaints concerned attention and executive functioning, the sustained visual attention and controlled auditory attention tests assessed their ability to maintain attention, selective attention, inhibition, and flexibility (15, 17), while forward and backward digit span tests evaluated working memory. Functional difficulty in everyday settings was measured according to DSM-5 criteria (2).

Test results for each child were anonymized, assigned codes, and sent to the research team through a secure online platform to be checked and recorded. The pediatricians referred their child patients to professionals specialized in the cognitive domains concerned, informing these specialists of the study protocol but not disclosing BMT-*i* results.

#### Administration of Reference Tests

The specialists performed their evaluations under the usual conditions of their work. Pediatricians gave the specialists a letter from the research team that specified the tests to be administered for each cognitive domain, chosen from among the commonly used, carefully validated test batteries indicated by the researchers. Recommended minimal WL skills (18, 19)—reading speed, accuracy, and comprehension, as well as spelling—were measured with the EVALEO (20), Exalang (21, 22), or BELO (23) speech-language batteries (Table 1). For MC, skills in numeric representation, mental math, and problem-solving were assessed with tests from three batteries adapted to children's school grades and, like the BMT-*i*, designed according to current neuropsychological models (24–26): TEDI-MATH (27), TEDI-MATH Grands (28), and Examath 8–15 (29). Recommended OL skills (30, 31) were assessed by speech-language pathologists using five standard language production and comprehension tests from the Evalo (32), Exalang (33), and EVALEO (20) batteries. Psychomotor or occupational therapists assessed HV abilities—handwriting, drawing, and visuospatial construction (34–36)—by having children (i) copy figures from the VMI (36) or NEPSY-II (37) test batteries; (ii) copy the Rey complex figure (38); (iii) write, measuring speed and quality with the BHK scale (39); and (iv) complete NEPSY-II (37) or WISC-V (40) cube construction tasks. To measure attention (17) and executive functioning (41) (AE), a neuropsychologist administered an IQ test (WISC-V) and the NEPSY-II Auditory Attention & Response Set subtest (37), along with others from the Conners Continuous Performance Test (CPT 3) (42), TAP/KiTAP (43), and Tea-ch (44) batteries that assess sustained attention and inhibition/flexibility. Working memory was gauged with the WISC-V Digit Span (40) or CMS Numbers (45) subtest. Functional impairment in daily life was evaluated with the Behavior Rating Inventory of Executive Function (BRIEF) (46).

### Analysis of Data

Data were analyzed for each of the five cognitive domains in question. For OL in particular, which concerned 60 children, the researchers also compared results from a short version of the BMT-*i* (an evaluation of lexical comprehension, syntactic production, and phonological quality, lasting about 10 min) with those for the five-skill speech-language assessment. In addition, they analyzed data for a homogeneous subgroup (46 out of the 60 children) whose members were all in the last year of French kindergarten (*grande section de maternelle*, or GSM) and had been evaluated using the same speech-language battery (Evalo).

Differences in school grades and reference test battery norms necessitated harmonization of scores before they could be compared. Thus, scores were converted to a three-point scale −0, or normal, if the cumulative percentage was >20%; 1, or low, if it was between 7 and 20%; and 2, or very low, if it was ≤7%—while preserving the correspondence between cumulative percentages, standard scores, and *z*-scores, in accordance with the American Academy of Clinical Neuropsychology consensus statement (47).

Score conversions were performed independently by pediatricians, for the BMT-*i*, and specialists, for reference batteries, in accordance with the study protocol. Each skill was rated on the basis of these BMT-*i* scores: a level of 2 was assigned when one of the scores was very low; 1 if multiple scores were low, and 0 in all other cases, including when only one score was low. Pediatricians then made one of three recommendations: specialized testing needed, if at least one of the skills had a very low rating (level 2); the need for specialized testing to be discussed, if multiple skills had low ratings (level 1); or no need for specialized testing, in any other case (level 0). Similarly, specialists categorized children as having a disorder (=2), moderately impaired (=1), or normal (=0).

For each cognitive domain, an independent expert (neuropsychologist or speech therapist) otherwise unassociated with the study performed a blind analysis of the pediatricians' and specialists' findings. The independent expert also rescored BMT-*i* and Rey complex figure copying tests. When the original score did not match the later one, a definitive score was assigned after discussion with the research team. For the assessment of attention in particular, explicit qualitative aspects observed in 4 of the children—e.g., transient fatigability, fluctuation, or slowness—influenced the interpretation of scores in the professional's conclusion, which was retained. For the purpose of calculating BMT-*i* sensitivity and specificity, scores indicating disorders (=2) or moderate deficits (=1) were grouped together, to distinguish both from normal (=0) scores.

### Statistical Analysis

Sensitivity, specificity, and both positive (LR+) and negative (LR–) likelihood ratios were calculated from the findings of the pediatricians (BMT-*i*) and specialists (reference test batteries). The desired sensitivity was >85%. The LR+ estimates the probability of correctly diagnosing a disorder when test results are positive. The supplemental diagnostic value of the test is low if the LR+ is between 1 and 2, intermediate if between 2 and 5, and high if >5. In contrast, the LR– estimates the probability of correctly rejecting diagnosis of a disorder when test results are negative. The supplemental diagnostic value of the test is low if the LR– is between 0.5 and 1, intermediate if between 0.2 and 0.5, considerable if between 0.1 and 0.2, and high if <0.1. The correlation between converted BMT-*i* and reference battery test scores was evaluated using the Matthews correlation coefficient (MCC), a derivative of the Pearson correlation coefficient for unbalanced populations (48). Agreement between the findings of pediatricians and specialists was measured with Cohen's kappa (κ), where values in the range of 0.21–0.40 indicate fair agreement; 0.41–0.60, moderate; 0.61–0.80, substantial; and 0.81–1.00, almost perfect agreement (49). Raw OL test scores for the GSM subgroup were compared using the Pearson correlation coefficient (*r*). The Pearson correlation coefficient was also calculated for comparison of raw scores from the BMT-*i* controlled auditory attention test and the NEPSY test.

## Results

### Characteristics of Study Population

Figure 1 illustrates the study inclusion process and provides a breakdown of the 250 complaints by cognitive domain. Only 14 of the 27 pediatricians initially identified were able to take part in the study. Spread across France −12 in cities and 2 in suburban areas −9 of them had private practices, while the remaining 5 worked in hospitals.

Of the 229 children preselected, 191 were included between March 31, 2019, and September 1, 2020, 28% of them presenting with complaints that concerned 2 or 3 cognitive domains. Due to parental refusal of consent or failure to follow the study protocol, 38 of the 229 children were excluded. For the 191 children included, 250 assessments for one of the five cognitive domains were conducted: 60 for WL; 40 for MC; 60 for OL, including the GSM subgroup of 46 children; 45 for HV; and 45 for AE. All children had a normal evaluation of their vision and hearing, and were searched for emotional disorders. Moreover, children included for a complaint affecting attention/executive functions did not receive any treatment during and between the two evaluations (BMT*-i* and specialized assessments). Teacher questionnaires were missing in 9% of the cases. Of the 250 assessments, 3% were missing a single skill score; 9%, DCDQ-FE (HV) data; and 15%, DSM-5 evaluations (AE). Table 2 summarizes population characteristics for each cognitive complaint. Children from bilingual (defined as one parent speaking a language other than French) and underprivileged families made up half (50%) of those in the OL category. Boys predominated in the MC (67%), HV (75%), and AE (73%) categories. Over half of the children included were undergoing reeducation, but the percentage varied by cognitive domain, ranging from 28% for OL to 72% for WL.

**Table 2:** Characteristics of study population.

**Skills**	**Written language (WL)**	**Mathematical cognition (MC)**	**Oral language (OL)**	**Handwriting, drawing and visuospatial construction (HV)**	**Attention and executive functioning (AE)**
Number of children	60	40	60	45	45
Mean age, months (range)	108.5 (78–155)	113 (87–155)	66.8 (55–82)	105.4 (62–155)	113.7 (85–164)
School grades concerned (%)	1 (5%) 2–4 (72%) 5–7 (23%)	2 (25%) 3–7 (75%)	KG (77%) 1 (23%)	KG−1 (11%) 2–4 (68%) 5–7 (21%)	2–3 (42%) 4–5 (38%) 6–7 (18%)
Time between tests, months (range)	0.35 (0–4)	0.53 (0–4)	0.13 (0–3)	1.4 (0–4)	1.3 (0–4)
Boys, % | Girls, %	55% | 45% not significant	67% | 33% *p* = 0.027	48% | 52% not significant	75% | 25% *p* = 0.0004	73% | 27% *p* = 0.0014
Proportion with perinatal background	8%	17.5%	7%	8.7%	9%
Bilingualism	28%	41%	50%	28%	27%
Underprivileged	21%	10%	50%	13%	7%
Receiving remedial support	72%	63%	28%	58%	53%
Scores from teachers' questionnaires^a^	2: 56% 1: 36% 0: 8%	2: 53% 1: 39% 0: 8%	2: 45% 1: 45% 0: 9%	2: 66% 1: 32% 0: 2%	2: 44% 1: 53% 0: 3%

*KG, kindergarten*.

a *0 = normal; 1 = moderate deficit; 2 = disorder*.

### Agreement Between Scores Assigned by Pediatricians and Specialists

**Figure 2:**
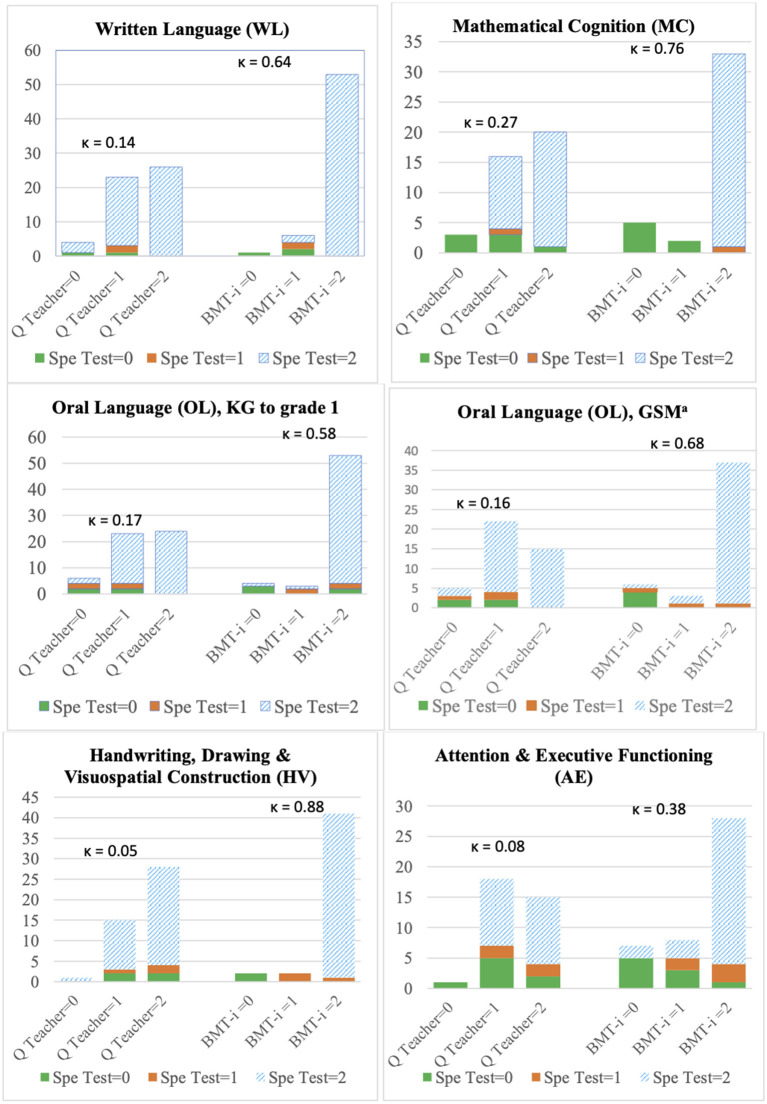
Agreement of BMT-i and teachers' questionnaires with specialized assessments. ^a^ GSM subgroup assessed by Evalo (*n* = 46); GSM, final year of French kindergarten (*grande section de maternelle*); κ, Cohen's kappa; KG, kindergarten; Q Teacher, teachers' questionnaire; Spe, specialized. Possible scores were 0 (normal), 1 (moderate deficit), 2 (disorder).

For each of the five cognitive domains considered, Figure 2 gives an overview of assessments made by specialists, pediatricians, and teachers, and Tables 3–**7** provide statistics measuring the correspondence between the findings of pediatricians and specialists.

**Table 3:** Written language: BMT-i sensitivity and agreement with specialized assessments.

**Global assessment for written language (*****n*** **=** **60)**	
**κ** (CI);^a^ probability	0.64 (0.38–0.91); *p* < 0.0001
Sensitivity^b^	1 (0.94–1)
Specificity^b^	0.33 (0.1–0.8)
LR+^b^	1.5 (0.7–3.3)
LR–^b^	0
MCC	0.57
**Breakdown by skill**	
**Skill**	**κ** ^a^
Reading time	0.71 (0.5–0.91); *p* < 0.0001
Reading errors (*n* = 58)^c^	0.57 (0.40–0.74); *p* < 0.0001
Reading comprehension (*n* = 59)^c^	0.61 (0.45–0.77); *p* < 0.0001
Dictation	0.58 (0.37–0.79); *p* < 0.0001

a *After conversion of scores to 0 (normal), 1 (low), or 2 (very low)*.

b *After conversion of scores to 0 (normal) or 1 (disorder or moderate deficit)*.

c *Lower values of n due to missing data*.

*CI, confidence interval; κ, Cohen's kappa; LR–, negative likelihood ratio; LR+, positive likelihood ratio; MCC, Matthews correlation coefficient*.

For WL (Table 3), analysis revealed substantial agreement between pediatricians' and specialists' scores (κ = 0.64), maximum sensitivity for the BMT-*i* (1), low specificity (0.33), and an intermediate MCC value (0.57). Values of κ were also satisfactory for each skill considered. They were higher for reading speed (0.71) and comprehension (0.61) than for accuracy (0.57) and spelling (0.58).

The speech-language pathologists diagnosed disorders for 91% of the participants, and pediatricians esteemed specialized testing was required for 88% of them (Figure 2). In 92% of the cases, teachers' questionnaires described complaints of major (49%) or moderate (43%) severity, in disagreement with the conclusions of the speech-language pathologist (kappa = 0.14). Speech-language assessments revealed very low reading speed (for 82% of children) and spelling (for 83%) levels, while the level of reading comprehension was low for only half of the children. A quarter of the children assessed as having disorders were not undergoing reeducation at the time of the study.

For MC (Table 4), analysis likewise showed considerable agreement between pediatricians' and specialists' scores (κ = 0.76), maximum sensitivity (1), high specificity (0.71), and a high MCC (0.82). Values of κ were moderate for conversion of numeric representations (0.49) and mental math (0.47). There was moderate to no agreement for problem-solving assessments, depending on the reference test battery used. It was low with TEDI-MATH Petits, moderate with Examath, and nil with TEDI-MATH Grands.

**Table 4:** Mathematical cognition: BMT-i sensitivity and agreement with specialized assessments.

**Global assessment for mathematical cognition (*****n*** **= 40)**			
κ (CI);^a^ probability		0.76 (0.54–0.98); *p* < 0.0001	
Sensitivity^b^		1 (0.90–1)	
Specificity^b^		0.71 (0.36–0.92)	
LR+^b^		3.5 (1.1–11.3)	
LR–^b^		0	
MCC		0.82	
**Breakdown by skill: overall and test-specific agreement**			
**Skill**	**Overall κ** ^a^	**Reference test**	**κ for reference test**
Conversion of numeric representations	0.49 (0.29–0.69); *p* < 0.0001	TEDI-MATH Petits (*n* = 12)	0.69 (0.37–1); *p* = 0.0017
		TEDI-MATH Grands (*n* = 18)	0.3 (0.02–0.58); *p* = 0.02
		Examath 8–15 (*n* = 10)	0.46 (0.06–0.87); *p* = 0.018
Mental math	0.47 (0.23–0.72); *p* < 0.0001	TEDI-MATH Petits (*n* = 12)	0.79 (0.46–1); *p* = 0.0005
		TEDI-MATH Grands (*n* = 18)	0.32 (0.0–0.78); *p* = 0.036
		Examath 8–15 (*n* = 10)	0.33 (0–0.76); *p* = 0.06, ns
Problem-solving (*n* = 38)^c^	0.13 (0–0.33); ns	TEDI-MATH Petits (*n* = 10)	0.31 (0–0.67); *p* = 0.04
		TEDI-MATH Grands (*n* = 18)	0 (0–0.25); ns
		Examath 8–15 (*n* = 10)	0.44 (0–0.96); *p* = 0.05

a *After conversion of scores to 0 (normal), 1 (low), or 2 (very low)*.

b *After conversion of scores to 0 (normal) or 1 (disorder or moderate deficit)*.

c *Lower value of n due to missing data*.

*CI, confidence interval; κ, Cohen's kappa; LR–, negative likelihood ratio; LR+, positive likelihood ratio; MCC, Matthews correlation coefficient; ns, not significant*.

Most children with MC complaints were diagnosed with disorders: 82% according to pediatricians using the BMT-*i*, and 80% according to specialists. In 92% of the cases, teachers' questionnaires described complaints of major (53%) or moderate (39%) severity (Figure 2); however, agreement with the conclusions of the speech-language pathologist was low (κ = 0.27).

The profile of deficits detected by the speech-language pathologist was mixed: mental math was severely affected for 65% of the cases; conversion of numeric representations, for 45%; and problem-solving, for 36%. At the time of the study, two-thirds of the children were undergoing speech-language reeducation for written language or mathematics.

Table 5 gives comparative statistics for OL assessments (*n* = 60). They indicate moderate agreement between the pediatrician's and specialist's global assessments for all five skills tested (κ = 0.58), high sensitivity (0.98), moderate specificity (0.60), and a moderate MCC value (0.66). Results are similar when we compare the short version of the BMT-*i* to the full speech-language assessment. However, for the GSM subgroup (*n* = 46), there was substantial agreement (κ = 0.68), and the sensitivity (0.98), specificity (0.75), and MCC value (0.73) were all satisfactory. For these 46 children, similar values were obtained when comparing results for the short BMT-*i* to those for the full speech-language assessment (sensitivity = 0.95; specificity = 1; MCC = 0.80).

**Table 5:** Oral language: BMT-i sensitivity and agreement with specialized assessments.

**Global assessment for oral language—five skills**^**a**^ **(*****n*** **=** **60)**^**b**^		
κ (CI);^c^ probability		0.58 (0.28–0.88); *p* < 0.0001
Sensitivity^d^		0.98 (0.90–0.997)
Specificity^d^		0.60 (0.23–0.88)
LR+^d^		2.5 (0.84–7.18)
LR–^d^		0.03 (0.0–0.2)
MCC^d^		0.66
**Breakdown by skill** ^**a**^		
**Skill**		**κ^c^**
Lexical comprehension		0.29 (0.08–0.50); *p* = 0.0016
Lexical production		0.28 (0.11–0.45); *p* = 0.0007
Syntactic comprehension		0.18 (0.02–0.33); *p* = 0.01
Syntactic production		0.26 (0.05–0.47); *p* = 0.0033
Phonology		0.35 (0.17–0.54); *p* < 0.0001
**Global assessment for oral language—three skills**^**a**^ **(*****n*** **=** **60)**^**b**^		
κ (CI);^c^ probability		0.52 (0.25–0.80); *p* < 0.0001
Sensitivity^d^		0.96 (0.88–0.99)
Specificity^d^		0.80 (0.38–0.96)
LR+^d^		4.8 (0.83–27.8)
LR–^d^		0.045 (0.01–0.19)
MCC		0.70
**GSM subgroup (*****n*** **=** **46):**^**e**^ **Global assessment for oral language—five** **skills**^**a**^		
κ (CI);^c^ probability		0.68 (0.38–0.97); *p* = 0.0001
Sensitivity^d^		0.98 (0.88–0.99)
Specificity^d^		0.75 (0.31–0.95)
LR+ ^d^		3.9 (0.72–21.3)
LR– ^d^		0.03 (0.004–0.24)
MCC		0.73
**Breakdown by skill**		
**Skill**	**κ** ^c^	**Comparison of raw scores**, ***r***
Lexical comprehension	0.44 (0.19–0.69); *p* < 0.0001	0.54 (0.29–0.72); *p* = 0.0002
Lexical production	0.32 (0.14–0.50); *p* = 0.0006	0.70 (0.59–0.77); *p* < 0.0001
Syntactic comprehension	0.16 (0–0.35); *p* = 0.04	0.54 (0.29–0.72); *p* = 0.0001
Syntactic production	0.32 (0.08–0.57); *p* = 0.0015	0.60 (0.38–0.76); *p* < 0.0001
Phonology	0.39 (0.18–0.60); *p* = 0.0001	0.62 (0.39–0.82); *p* < 0.0001
**GSM subgroup (*****n*** **=** **46):**^**e**^ **Global assessment for oral language—three** **skills**^**a**^		
κ (CI);^c^ probability		0.64 (0.37–0.91); *p* < 0.0001
Sensitivity^d^	0.95 (0.84–0.99)	
Specificity^d^	1 (0.91–1)	
LR+^d^	^f^	
LR–^d^	0.048 (0.012–0.18)	
MCC	0.80	

a *Five-skill BMT-i oral-language assessment evaluated lexical comprehension and production, syntactic comprehension and production, and phonology, while three-skill version solely evaluated lexical comprehension, syntactic production, and phonology. Results of three-skill BMT-i assessments were still compared with five-skill reference battery tests*.

b *Of the 60 children tested, 3% were younger kindergartners; 90% were in GSM (grande section de maternelle), the final year of kindergarten; and 7% were first graders*.

c *After conversion of scores to 0 (normal), 1 (low), or 2 (very low)*.

d *After conversion of scores to 0 (normal) or 1 (disorder or moderate deficit)*.

e *The reference test battery used for all 46 children in the GSM subgroup was Evalo. CI, confidence interval; GSM, final year of kindergarten (grande section de maternelle); κ, Cohen's kappa; LR–, negative likelihood ratio; LR+, positive likelihood ratio; MCC, Matthews correlation coefficient; r, Pearson correlation coefficient*.

f *Not calculable (1 – specificity = 0)*.

If we consider the study population as a whole, κ values for each skill were low. Yet, in the GSM subgroup, BMT-*i* and reference test scores were strongly correlated for each of the five skills assessed (*r*: 0.54–0.70; *p*: < 0.0001–0.0002).

The majority of both the study population and the GSM subgroup were assessed as having OL disorders by the pediatrician (88%) and the specialist (85%) alike, though only 28% were following a course of speech-language reeducation at the time of the study. In 89% of the cases, the teachers' questionnaires described complaints of major (45%) or moderate (42%) severity, and they were not sufficiently in agreement with the speech-language pathologist's assessment (κ = 0.17) (Figure 2). The latter revealed a mixed profile of lexical or syntactic deficits for 75% of the cases and phonological deficits for 40%.

Table 6 presents data for the HV domain. Here there was an outstanding level of agreement between the pediatrician's and specialist's assessments (κ = 0.88). The BMT-*i* sensitivity, specificity, and MCC statistics all had maximum values (=1). Levels of agreement nonetheless differed by skill: they were high for handwriting, moderate for the Rey complex figure, and low for the visuospatial construction tests. There was no agreement for copying of simple figures.

**Table 6:** Handwriting, drawing, and visuospatial construction: BMT-i sensitivity and agreement with specialized assessments.

**Global assessment for handwriting, drawing, and visuospatial** **construction (*****n*** **=** **45)**	
κ (CI);^a^ probability	0.88 (0.65–1); *p* < 0.0001
Sensitivity^b^	1 (0.92–1)
Specificity^b^	1 (0.34–1)
LR+^b^	^c^
LR–^b^	0.0
MCC	1
**Breakdown by skill**	
**Skill**	**κ^a^**
Copying simple figures	0.12 (0.10–0.035); *p* = 0.13, not significant
Copying complex figure	0.43 (0.18–0.68); *p* = 0.0002
Handwriting	0.76 (0.51–1); *p* < 0.0001
Visuospatial construction	0.34 (0.16–0.52); *p* < 0.0001

a *After conversion of scores to 0 (normal), 1 (low), or 2 (very low)*.

b *After conversion of scores to 0 (normal) or 1 (disorder or moderate deficit). CI, confidence interval; κ, Cohen's kappa; LR–, negative likelihood ratio; LR+, positive likelihood ratio; MCC, Matthews correlation coefficient*.

c *Not calculable (1 – specificity = 0)*.

Most pediatrician's (91%) and specialist's (89%) assessments diagnosed disorders. For 98% of all cases, teachers' questionnaires indicated complaints of major (62%) or moderate (36%) severity and were in disagreement with the specialist's assessment (κ = 0.05) (Figure 2). DCDQ-FE responses showed that developmental coordination disorder was suspected for 63% of the children, but there was a lack of agreement with the assessments of the pediatrician (κ = 0.01; not significant) and the specialist (κ = 0.02; not significant). Children's HV profiles varied, but handwriting deficits were detected in 80% of the initial assessments and 83% of the specialized assessments. One out of two children had difficulties with the complex figure copying task.

The systematic psychometric evaluation in AE domain confirmed the absence of mental disability: the average values of the Verbal Comprehension Index (VCI), Fluid Reasoning Index (FRI) and Visual Spatial Index (VSI) were 105, 104, and 100, respectively. None of the children had any of those indexes below 81. AE disorders were reported for 67% of the BMT-*i* and of the specialized test (Figure 2). The global AE assessments of the pediatrician and the neuropsychologist (Table 7) were fair in agreement (κ = 0.38), sensitivity high (0.91), and specificity (0.56) and MCC value (0.49) both modest. On the other hand, normal BMT-*i* results suggested that a normal neuropsychological assessment was fairly likely (LR– = 0.16). There was a low but acceptable level of agreement for the sustained attention and flexibility/inhibition tests. For the selective attention and digit span tests, the level of agreement was insufficient.

**Table 7:** Attention and executive functioning: BMT-i sensitivity and agreement with specialized assessments.

**Global assessment for attention and executive functioning (*****n*** **=** **43)**^c^		
κ (CI);^a^ probability		0.38 (0.14–0.62); *p* = 0.0004
Sensitivity^b^		0.91 (0.77–0.97)
Specificity^b^		0.56 (0.27–0.81)
LR+^b^		2.1 (1.0–4.4)
LR–^b^		0.16 (0.04–0.5)
MCC		0.49
**Breakdown by skill**		
**Skill**	**κ** ^a^	**Comparison of raw** **scores**, ***r***
Sustained attention (*n* = 43)	0.33 (0.13–0.53); *p* = 0.0009	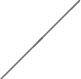
Selective attention (*n* = 45)	0.19 (0.0–0.39); *p* = 0.023	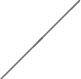
Flexibility/inhibition (*n* = 45)	0.30 (0.1–0.52); *p* = 0.0006	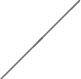
BMT-*i* “conflict” task vs. NEPSY-II Attention Response set (*n* = 43)	0.35 (0.14–0.56); *p* = 0.006	0.71 (0.53–0.84); *p* < 0.0001
Working memory	0.18 (0–0.40); *p* = 0.07, not significant	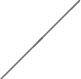

a *After conversion of scores to 0 (normal), 1 (low), or 2 (very low)*.

b *After conversion of scores to 0 (normal) or 1 (disorder or moderate deficit)*.

c *Lower value of n due to missing data*.

*CI, confidence interval; κ, Cohen's kappa; LR–, negative likelihood ratio; LR+, positive likelihood ratio; MCC, Matthews correlation coefficient; r, Pearson correlation coefficient*.

In 97% of the cases, teachers' questionnaires revealed attentional complaints of major (44%) or moderate (53%) severity, though there was no agreement with the specialist's assessment (κ = 0.08) (Figure 2). DSM-5 criteria were met (≥6 symptoms) in 82% of all cases for inattention and in 47% of the cases for hyperactivity/impulsivity, but there was no agreement with the overall neuropsychological assessment (κ: 0.01 and 0.01, respectively; not significant). In five children (10%), neither of the two DSM-5 scales confirmed the diagnostic criteria for ADHD but four of the five had abnormal results with both the BMT-*i* and the specialized evaluations. For the BRIEF assessments, the mean Global Executive Composite was at the disorder threshold (T-score: 70) and the mean Metacognition Index was close to it (T-score: 69). These values did not vary with the findings of the neuropsychological assessments.

Children's attentional profiles were very diverse. Disorders of selective attention were identified for 38% of the cases; of sustained attention, for 35%; and of flexibility/inhibition, for 49% of the specialized assessments. The correlation between NEPSY raw scores (Response set) and BMT-*i* auditory attention results (“conflict” task) was highly significant [*r* = 0.71 (0.53–0.84); *p* < 0.0001].

## Discussion

For our cohort of children with cognitive complaints, we have reported the sensitivity of the BMT-*i* in the domains concerned (2, 9–11) and its agreement with the reference test batteries used by specialists. This study was only possible due to the earlier validation of the BMT-*i* with a vast cross-sectional sample of French school children with no complaints (15). The large proportion of children receiving no remedial support despite difficulties detected during the initial examination underscores the value of screening with the BMT-*i*. The fact that 28% of children were struggling in more than one cognitive domain also argues for a single, comprehensive battery to screen for difficulties in multiple skill areas (15).

Our findings confirm the high sensitivity (0.91–1) of overall BMT-*i* assessments compared with specialized batteries, for each of the domains considered. Furthermore, the likelihood of normal BMT-*i* results accurately predicting normal results for a specialized assessment was considerable. Specificity varied across domains (0.33–1): high for MC and HV; moderate for OL and AE; and low for WL. The likelihood that BMT-*i* results indicating a deficit would accurately predict the identification of a disorder through a specialized assessment was fair for MC, HV, and OL; moderate for AE; and low for WL. The level of agreement between global BMT-*i* assessments and specialists' assessments was excellent for MC and HV; substantial for WL, as well as for OL in the GSM subgroup; but lower for AE. Thus, the BMT-*i* offers a level of performance expected of first-line screening instruments whose aim is to identify the majority of children in need of referral to a specialist.

The extent to which BMT-*i* and specialized assessments agreed on a test-by-test basis varies between cognitive domains. Agreement was lowest for arithmetic problems (MC), syntactic comprehension (OL), copying of simple figures (HV), and selective attention (AE).

A consensus exists on the need for validated tools suitable for each cognitive domain—not only to identify students who are struggling, but also to determine the profile and magnitude of their cognitive strengths and weaknesses, and to monitor progress made (3–5, 50). The BMT-*i* also provides such information for the particular skills affected. For example, by profiling and gauging the severity of deficits in individual WL skills, the BMT-*i* can help choose the appropriate next step (18), be it an urgent referral for a specialized assessment in the event of a severe deficit or one affecting reading comprehension (19), or an educational intervention in the event of a deficit in reading speed alone (51). For MC, from kindergarten to middle school, the BMT-*i*, in accordance with current neuropsychological models (24–26, 52), offers a first-line assessment of numeric representation and arithmetic skills, which are weaker among French students (53). With regards to OL, use of the shorter three-test BMT-*i* allows for efficient and reliable detection of language deficits among older kindergartners (GSM). Interviews alone are not as effective in identifying such deficits (54), especially in underprivileged settings (30, 31). HV test results complement the information provided by the DCDQ-FE, which is limited to motor deficits (16), permitting detection of handwriting, drawing, and visuospatial impairments seen in children with developmental coordination disorder (55) or as isolated conditions (56). The BMT-*i* affords a comprehensive vision of the various components of writing (speed, handwriting, and spelling), to help define a remedial program when dysgraphia is present (57). In the case of AE difficulties, a preliminary formal computerized assessment of cognitive functions using the BMT-*i* supplements' data from questionnaires, evaluates the severity of any associated academic deficits (58), and can affirm the need for a neuropsychological assessment, which is recommended in the presence of AE complaints (17, 41, 58).

International recommendations propose supplementing the standard psychometric evaluations—e.g., Wechsler (40), NEPSY (37), and KABC II (59) scales—with test batteries measuring academic skills. There are numerous English-language test batteries of this sort, including the Wechsler Individual Achievement Test–Second Edition (WIAT-II) (60), Wide Range Achievement Test−5th Edition (WRAT5) (61), Woodcock-Johnson IV Tests of Early Cognitive and Academic Development (ECAD) (62), and the Kaufman Test of Educational Achievement–Second Edition (KTEA-II) (63), taken to be the standard instruments. With the exception of the WIAT-II, normed with a limited sample of francophone Canadians, none has been calibrated for a French-speaking population (64). Furthermore, as these batteries are used in combination with a psychometric evaluation, they are better described as integral components of long and costly specialized assessments than as screening tools. While the EPOCY (65), based on the KTEA-II, is a French-language battery, it only evaluates academic skill levels. None of these instruments is computerized, nor do they allow for simultaneous evaluation of skills and underlying cognitive functions. Conversely, the BMT-*i* is the only general standardized modular instrument for first-line assessment of the strengths and weaknesses of children's academic skills and cognitive functioning.

There are, however, several limitations of our study. Firstly, our sample solely consisted of children with cognitive complaints. A study simultaneously considering cohorts of children with and without complaints would undoubtedly have been more balanced, but obtaining reliable normative data for a representative population without prior complaints was a crucial prerequisite for a study of children with complaints (48). Hence, this external validation cannot be regarded as a cohort study with systematic evaluation of all possibly affected domains in LDs nor as a conclusive diagnostic study. Indeed, the population studied included both children seen for the first time and children already taken into care but needing further specialized evaluation. Secondly, we observed a high frequency of parental bilingualism and of OL difficulties—though this is consistent with data from the literature (30, 31)—as well as a disproportionately large number of boys with attentional and HV disorders (2, 66). Thirdly, in contrast with the United States, there is a lack of consensus in France regarding which specialized test batteries are to be preferred. This drove us to use multiple reference tests for each cognitive domain, selecting them on the basis of the quality of their validation (67). The specialized assessments included, in addition to the basic skills that were compared to those of the BMT-i, many other tests assessing more precisely the different cognitive functions in order to build a therapeutic project adapted to the child. A final limitation of the study concerns the low level of agreement between certain skills in the domains of CM (problem-solving), LO (syntactic comprehension), GR (copying simple figures), and AE (selective attention and working memory). Several factors might explain this, such as the diversity of reference tests; differences between tasks, as was the case for syntactic comprehension and problem-solving; or different test-taking modes, i.e., computer vs. pencil and paper (for attention tests).

In conclusion, the BMT-*i* is a test battery for first-line screening of LDs in children. To our knowledge, no other tool for initial assessment of all cognitive domains concerned is available (15, 47). The various situations in which children are affected by Learning Disorders open many other fields of future studies and possible applications using the battery. The BMT-*i* could be used for initial assessment when educational intervention methods fail to improve the child's learning difficulties (5, 11). It could also be used to detect sequelae of acquired cerebral or perinatal lesions or as a first cognitive assessment in others groups of neurodevelopmental disorders, such as intellectual disabilities or autism spectrum disorders. The BMT-*i* is quickly administered, sensitive, easy to interpret, and affordable for all. It is an easy, low-cost means of identifying children requiring referral to specialists for more precise diagnoses and appropriate remediation (3–5, 8, 11, 13).

## Data Availability Statement

The raw data supporting the conclusions of this article will be made available by the authors, without undue reservation.

## Ethics Statement

The studies involving human participants were reviewed and approved by CPP 2018-A-O1870-55 Comité de protection des personnes Sud MEDITERRANEE CHU de Cimiez, CS 91179-06003 NICE CEDEX1. Written informed consent to participate in this study was provided by the participants' legal guardian/next of kin.

## Author Contributions

CB, CJ, and T-NW designed the study. MT, SG, and AMi selected the reference tests. CB, SG, and MT led the study and collected data. CB and CJ performed analyses. AMi and MT discussed the results. CB wrote the manuscript. CJ, AMu, and T-NW revised the manuscript. All authors contributed to the article and approved the submitted version.

## Funding

The conception and initiation of the project was supported through grants from ARS: *Agence Régionale de Santé* (Regional Health Agency) from the following regions: Auvergne-Rhône-Alpes (2018-DA-126), Bourgogne-Franche Comté (ARSBFC/2018//841), Bretagne (2019/DIS/01A), Centre Loire, Occitanie (1.2.22/C258), Provence-Alpes Côte d'Azur. An additional grant was provided by the Fondation d'entreprise Hermès, through the involvement of the local network Recital 63 devoted to NDD in Puy-de-Dôme, France.

## Conflict of Interest

The authors declare that the research was conducted in the absence of any commercial or financial relationships that could be construed as a potential conflict of interest. The handling editor declared a shared affiliation with one of the authors AMu.

## Publisher's Note

All claims expressed in this article are solely those of the authors and do not necessarily represent those of their affiliated organizations, or those of the publisher, the editors and the reviewers. Any product that may be evaluated in this article, or claim that may be made by its manufacturer, is not guaranteed or endorsed by the publisher.

## Abbreviations

AE: attention and executive functioning
BMT-*i*: Batterie Modulable de Tests informatisée (computerized Adaptable Test Battery)
DCDQ-FE: European-French Developmental Coordination Disorder Questionnaire
HV: handwriting, drawing, and visuospatial construction
κ: Cohen's kappa
LD: learning disability
LR: likelihood ratio
MC: mathematical cognition
MCC: Matthews correlation coefficient
OL: oral language
WL: written language.
